# Measured and simulated mechanical properties of additively manufactured matrix-inclusion multimaterials fabricated by material jetting

**DOI:** 10.1186/s41205-023-00201-y

**Published:** 2024-02-02

**Authors:** Erik Kornfellner, Markus Königshofer, Lisa Krainz, Arno Krause, Ewald Unger, Francesco Moscato

**Affiliations:** 1 Center for Medical Physics and Biomedical Engineering, Medical University of Vienna, Vienna, Austria; 2 Ludwig Boltzmann Institute for Cardiovascular Research, Vienna, Austria; 3 Austrian Cluster for Tissue Regeneration, Vienna, Austria

**Keywords:** Additive manufacturing, Material jetting, Multimaterials, Matrix-inclusion-composite, Young’s modulus, Poisson’s ratio

## Abstract

Modern additive manufacturing enables the simultaneous processing of different materials during the printing process. While multimaterial 3D printing allows greater freedom in part design, the prediction of the mix-material properties becomes challenging. One type of multimaterials are matrix-inclusion composites, where one material contains inclusions of another material. Aim of this study was to develop a method to predict the uniaxial Young’s modulus and Poisson’s ratio of material jetted matrix-inclusion composites by a combination of simulations and experimental data.

Fifty samples from commercially available materials in their pure and matrix-inclusion mixed forms, with cubic inclusions, have been fabricated using material jetting and mechanically characterized by uniaxial tensile tests. Multiple simulation approaches have been assessed and compared to the measurement results in order to find and validate a method to predict the multimaterials’ properties. Optical coherence tomography and microscopy was used to characterize the size and structure of the multimaterials, compared to the design.

The materials exhibited Young’s moduli in the range of 1.4 GPa to 2.5 GPa. The multimaterial mixtures were never as stiff as the weighted volume average of the primary materials (up to $33.9\%$ softer for 45% *RGD8530-DM* inclusions in *VeroClear* matrix). Experimental data could be predicted by finite element simulations by considering a non-ideal contact stiffness between matrix and inclusion ($F_A=2.2\frac{TN}{m^3}$ for *RGD8530-DM*, $F_A=4.8\frac{TN}{m^3}$ for *RGD8430-DM*), and geometries of the printed inclusions that deviated from the design (rounded edge radii of $r=220\,\upmu$m). Not considering this would lead to a difference of the estimation result of up to $612\,$MPa (44%), simulating an inclusion volume fraction of 45% *RGD8530-DM*.

Prediction of matrix-inclusion composites fabricated by multimaterial jetting printing, is possible, however, requires a priori knowledge or additional measurements to characterize non-ideal contact stiffness between the components and effective printed geometries, precluding therefore a simple multimaterial modelling.

## Introduction

Additive manufacturing, or 3D printing, systems can do much more than just rapid prototyping. While these systems are often used in industry and the arts to quickly create and improve the design of parts, they are also being used to manufacture parts that would otherwise be difficult or impossible to produce using conventional techniques.

In medicine, additive manufacturing has already reached the point where models can be manufactured directly from the patient image data to train surgeons and to plan complex operations, thus enabling better treatment methods to be developed. Intra-operative surgical tools, as well as implants, have become the new gold standard in medical fields such as orthopedics [1, 2] and dentistry [3, 4].

However, materials ideally suited for medical applications should be as close as possible to natural ones, which come in many different forms and many different properties. Often it is indeed hard to find a singl synthetic material with the same mechanical properties when it comes to replacing or mimicking a human tissue or manufacturing implants and tissue engineering scaffolds [5]. This does not necessarily have to involve materials that are actually used in patient treatment. Most aspiring doctors are trained using body donations, if they even have the opportunity to practice procedures under conditions that approximate real-life scenarios. Since the quality of surgery is significantly influenced by the preparation and training of the surgeon, there is a growing effort to create models for these purposes. Naturally, the closer 3D-printed materials can mimic real tissue, the better.

Modern additive manufacturing allows to fabricate material composites in great variety, including some that resemble natural materials more closely than others. Mix- or multimaterials can be fabricated additively by different means, either by printing two materials close to each other or by mixing the raw materials before depositing them onto the building plate [6].

Among the different multimaterial possibilities, matrix-inclusion composites are of special interest for this study. Matrix-inclusion composites are materials consisting of one continuous phase, the matrix, containing dispersed inclusions from secondary materials. There are many other possible structures such as discontinuous phase composites or interpenetrating composites with different geometries [7]. As the number of phases increases, so does the complexity and range of possible structures. Human tissues, such as bone, provide examples of how such a composite can be organized into multiple hierarchical levels, each containing phases of different geometries and materials [8–11]. However, in this study single unconnected inclusions of one phase embedded in a matrix material were considered. Even for this type of composite, many possibilities exist because the shape and distribution of the included phases can be varied.

It is however important to notice that materials can also be mixed during material jet printing. The term digital material has become established for such print products. In this process, two or more primary materials are mixed by the printer to fill one voxel. On the size scale of the print resolution, this results in a homogeneous material [12, 13].

A wide variety of different printing processes to additively manufacture structures, each with their appropriate materials and applications, exists [14, 15]. Multimaterial jetting has been considered here, as this is one key process to fabricate material composites. Hereby photopolymer droplets are dispensed from the print head and subsequently cured with a UV lamp. The resulting mechanical properties of the printed part depend strongly on the used primary materials [16, 17] print orientation [18], and photopolymer curing degree [19–21].

Numerous studies have already investigated composites involving micro- or nano-particles, emphasizing the strategic alignment of these particles within the material to achieve specific properties [22–24]. In the context of material jetting, however, the inclusions are printed, enabling precise design of their shape and orientation. It should be noted that the inclusions, in this case, are larger than nano-particles, in a size that is fabricable with the printer resolution ($>40\,\upmu$m for $600\,$dpi), and also consist of photopolymer similar to the matrix material.

To alter the mechanical properties of the finished part multimaterial printing comes into play, allowing to fine-tune material mixtures and printed geometries to achieve the desired properties [25, 26]. Prior studies investigated the mechanical properties of multimaterial jetting, by assessing the influence of curing grade and associated hardness of additively manufactured parts [27] or its chemical properties [28]. It has already been shown by Salcedo et al., that test specimens consisting of multiple materials can be simulated by finite element modelling (FEM) [26]. However, only one large inclusion was included in the specimen and strain differences between simulation and experiment were found at the interface of the different materials. This shows the necessity for better modelling of the interfaces, especially in the case of matrix-inclusion composite which involves numerous individual inclusions. Other studies investigated estimations of the tensile strength of material jetted multimaterials, where the blending was performed on a voxel-wise basis [13, 29, 30]. Various models have been employed to describe mixed material jetted materials, however, none of them deals with a matrix-composite structure with inclusions ordered in unit cells.

Although there are already approaches to model matrix-inclusion composites [31, 32], these methods have not been compared extensively with experimental results from additively manufactured parts. Also, a generalized design approach is missing, to predict and plan multimaterials to achieve specific material properties.

Several studies have reported that the different material phases mix at their interface and form an intermediate layer [26, 29, 30, 33]. The most comprehensive examination was conducted by Zorzetto et al., in which material transitions of material jetted specimens were determined. Using nanoindentation, interface transitions up to $150\,\upmu$m in width were identified [33]. Salcedo et al. observed these blurred material transitions both in the scanning electron microscope and the light microscope. However, he did not incorporate them into his simulation model, and his simulation deviates from the experimental results precisely at the material transitions [26]. Bezek et al. utilized the blurred interfaces to blend the two materials voxel-wise, aiming to create a smoother transition between the two materials [30]. However, no previous study has investigated how this affects the properties of the overall material and how these effects can be incorporated into a simulation model.

The current study focuses on matrix-inclusion composites by using multimaterial jetting. The composite is of a scale that the inclusions can be observed with the bare eye (0.1 to $1\,$mm), with the composite becoming nevertheless similar to a homogeneous material in case e.g. of medical models, printed at a scale typically larger than $10\,$mm.

In particular, this study examines the fabrication of the samples, their mechanical characterization, and mathematical simulation approaches for predicting composite mechanical properties as a function of the inclusion volume fraction. In addition, the accuracy of the fabricated geometries and the printing process itself are discussed in terms of their implications for improving the predictive ability of simulations.

This article is structured such that it begins by elucidating the methods used for 3D printing the test specimens. Followed by a description of the geometric characterization and the mechanical testing procedure. Subsequently, the two simulation methods are explained. In the next section, the results about to the aforementioned steps are presented, before concluding with a discussion of these findings.

## Materials and methods

Samples were produced with an *Connex3 Objet500* polyjet printer (Stratasys Ltd., Minnesota, USA), from the raw materials *VeroClear*, *VeroPureWhite*, *TangoPlus* and *TangoBlackPlus*, all of them are acrylic-based photopolymers. The printer was operated with the software *Objet Studio Ver. 9* (Stratasys Ltd., Minnesota, USA). The material jet printer has a lateral resolution of $600\,$dpi and used a layer thickness of $30\,\upmu$m, therefore the voxels have a dimension of $42.3\times 42.3\times 30\,\upmu$m.

After the printing, the samples have been cleaned and dried. All samples have been processed with the so-called “matte” setting, covering the whole sample in support material and therefore producing a uniform surface on the top and bottom side of the sample. Alternatively, there is the “glossy” setting, where no support material is applied, resulting in different surface structures on the sample’s upper side and the bottom side facing the build platform. However, this machine setting does not affect the interfaces between matrix and inclusions.

Sample sets of 5 specimens each are considered, according to ISO 527 [34]. By fabricating *VeroPureWhite* together with *TangoBlackPlus*, the digital material *RGD8530-DM* can be created. Multimaterial samples with 3 different inclusion volume fractions $f_{inc}=$10%, 30% and 45% were designed using *VeroClear* as the matrix material and *RGD8530-DM* for the inclusions. From preliminary prints and literature is known, that the material phases combine with a certain layer thickness at the interface. Therefore samples with higher volume fractions have not been printed to avoid unintentional connection of the inclusions, since the aim of the study was to investigate the matrix-inclusion composites. Also, cubical inclusions were chosen, since other inclusion geometries would connect to each other at lower volume fractions. For example, spheres would have a diameter of $1\,$mm at a volume fraction of $f=52.4\%$ in a $1\,$mm^3^ cubical unit cell.

The 3 sets of multimaterials have been printed together with the 2 sets of pure primary materials in a single print job. All samples were printed with the same orientation, the long axis of the specimen in the direction of the print head movement.

A second print job was done using the same setup as stated before, but with *RGD8430-DM* as the inclusion material, which is fabricated using *VeroPureWhite* and *TangoPlus*. Altogether 10 sets with a total of 50 samples were prepared and tested.

These materials were selected based on their established miscibility and adhesive characteristics at the inclusion-matrix interface. For higher contrast in the OCT and visibility of the inclusions in the microscope, a transparent matrix and opaque inclusion materials were chosen. All samples were stored and dried for three days between printing and mechanical testing.

### Specimen geometry

The specimen geometry used is the *“Type A”* multipurpose test specimen from ISO 3167 [35], as required by ISO 527 [34], often referred to as dogbone. All specimens contain inclusions in a regular pattern, arranged in cubic unit cells, where the inclusion is in the center of the unit cell. Each unit cell has a side length of 1 mm, consisting of matrix material on the outside and an inclusion in its center. The placement in the tensile specimen was done in a way, that in the cross section of the gauging area fitted $10\times 4$ complete unit cells. The dimensions of the inclusions have been chosen in a way, that the inclusions account for a certain proportion of the total volume, for example, a cubic inclusion with an edge length of of $464\,\upmu$m would have a volume fraction of 10% in a $1\,\textrm{mm}^3$ unit cell.

### Measurements

The measurement of the mechanical properties was performed according to ISO 527 [34]. The specimens were fixed in the test stand *Messphysik BETA 10-2,5* (Messphysik GmbH, Fürstenfeld, Austria) and then pulled apart with a feed rate of $0.75\,\frac{\textrm{mm}}{\textrm{min}}$, which was increased to $3\,\frac{\textrm{mm}}{\textrm{min}}$ after 1.5 mm of displacement. The pretension was 1N and the length and width of the specimen were observed with an *OS-65D* video extensometer (Mintron Enterprise Co., Ltd., Taipei, Taiwan). The values were captured at $50\,$Hz. The Young’s modulus was evaluated at strains from $\varepsilon _1 = 0.05\%$ to $\varepsilon _2 = 0.25\%$ in the linear range of the material from the measured force. The start and end values of the measurement were determined by linear regression in the measuring range. For lateral strain, a 20-frame moving average was applied before calculating Poisson’s ratio to reduce scatter.

To characterize the dimensions of inclusions in the printed specimen, three optical methods have been employed: optical microscopy and profilometry as well as optical coherence tomography. Microscope images were taken with the *Levenhuk DTX 90* (Levenhuk, Inc., Florida, USA) and *VHX-7000* (Keyence International, Mechelen, Belgium) digital microscopes. Profilometer measurements of the sample surface were made with the optical profilometer *Keyence VR-5000* (Keyence International, Mechelen, Belgium).

The samples have been inspected with a custom-made swept-source optical coherence tomography (OCT) system operating at 1300 nm with a bandwidth of 30 nm [36]. This wavelength allows to easily image through the whole phantom thickness. A field of view (FoV) of $10\times 10\,$mm was acquired, the B-scan direction being roughly perpendicular to the printing direction of the phantom. After standard OCT post-processing, the 3D images were rotated to have the inclusion pattern coalign with the imaging axis. An average intensity projection over a depth of 812$\,\upmu$m (see Fig. 7), covering the first inclusion layer, was used to estimate the edge radius of the inclusions.

For a closer look at the microstructure, a second, microscopic spectral domain OCT was used, working at $800\,$nm with a bandwidth of 200 nm [37].

### Simulation

Two approaches for the simulation of the mechanical behavior of multimaterial properties were used: numerical finite element analysis (finite element method, FEM) was performed with *ANSYS* (ANSYS, Inc, Canonsburg, USA) and analytical continuum mechanical simulations, derived from the Mori-Tanaka scheme [38], performed with *MATLAB* (The MathWorks, Inc., Natrick, USA).

#### Finite element analysis

Although additively manufactured parts have anisotropic mechanical properties [18, 39, 40], the FEM simulations have been performed with an isotropic material model as an approximation for the uni-axial tensile tests. The primary material properties (Young’s modulus and Poisson’s ratio) have been obtained from the measurements. The volume fraction of the inclusion was varied in $5\%$ steps. Input for the design was the side length of the inclusion, while the edge radius was considered to achieve the correct volume fractions. To model the interfaces between inclusion and matrix, a bonded contact with an absolute value for normal stiffness was provided.

**Fig. 1:**
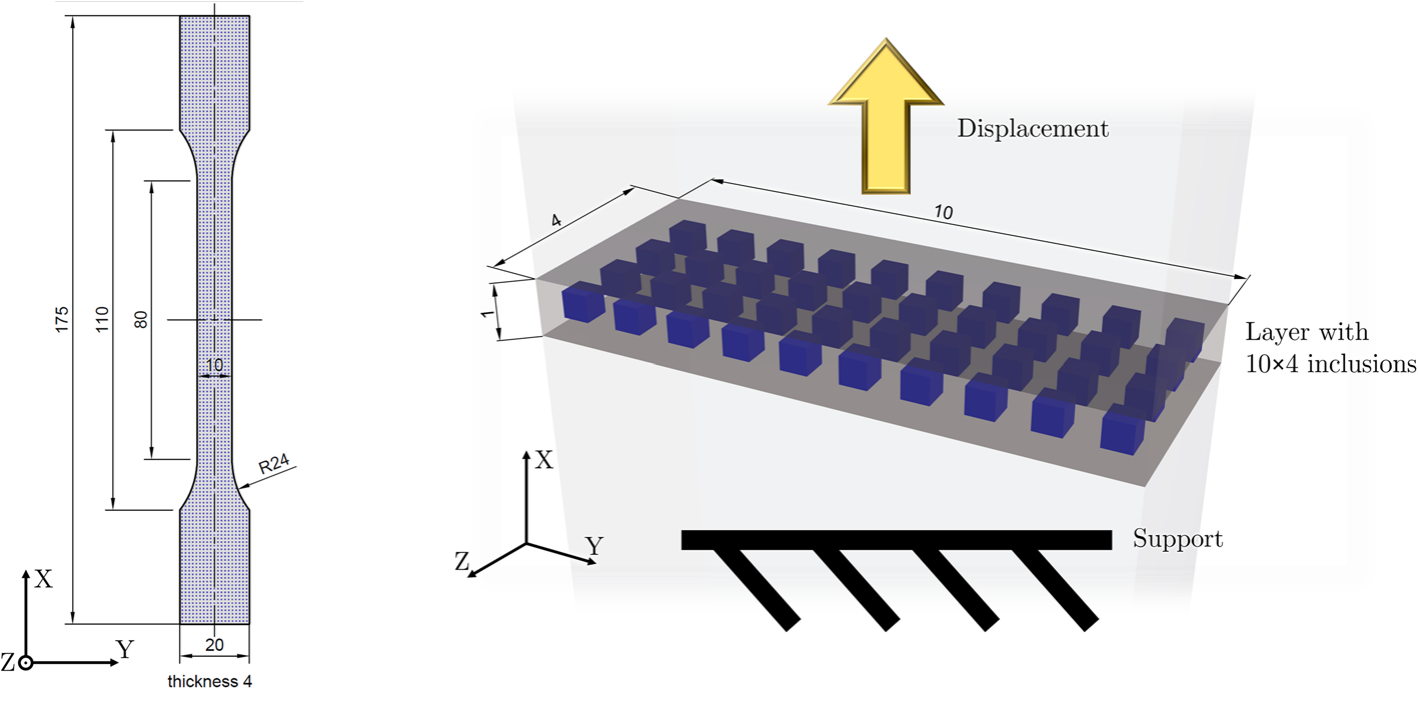
Left: Drawing of the specimen with inclusions. During the measurements, it was loaded in X-direction. A layer from the center was cut out and used for simulation as shown on the right side. Right: Render of the simulated layer with $10\times 4$ inclusions. The bottom side is fixated in X direction and the upper surface is displaced to reach a strain of $\varepsilon =0.25\%$

Mimicking the test setup, the FEM analysis was performed with $10\times 4$ unit cells of one millimeter cell length, which equates to one layer of unit cells within the tensile test specimen, as shown in Fig. 1. There are no interfaces between the individual unit cells since all matrix phases have been merged to one single geometry. The geometries were created with the ANSYS tool “DesignModeler”, the definition of boundary conditions, meshing and simulation were done with the ANSYS tool “Mechanical”.

A strain of $\varepsilon =0.25\%$ was applied transverse to the unit cell plane, similarly to the range applied during experimental testing, while the other side was fixated in the direction along the strain. The average stress in the direction of the elongation has been recorded to calculate the macroscopic Young’s modulus from the simulated uniaxial stress and the preset strain. The Poisson’s ratio of the material mixtures was evaluated by measuring the average displacement of the geometrical side planes compared to the preset strain. The calculation of the material parameters from this simulation setup was validated with pure materials.

Adaptive meshing size was used with a maximum element size of $300\,\upmu$m, using the finest resolution from the standard meshing tool, using about $2.1\cdot 10^6$ to $5.9\cdot 10^6$ mesh nodes and $1.4\cdot 10^6$ to $3.7\cdot 10^6$ mesh elements, depending on the inclusion geometry. The triangular meshes were checked for convergence. A higher number of mesh nodes was required for inclusions with very high volume fraction ($f_{inc}\rightarrow 85\%$) due to the larger material interface and for very small inclusions ($f_{inc}\rightarrow 5\%$) because of the potential for more movement in the matrix. The contact between matrix and inclusions was defined as bonded, but with different normal stiffnesses $F_A$. The average stress in the direction of the elongation has been recorded to calculate the Young’s modulus. Three values for this stiffness were evaluated as well as idealized bonding ($F_A\rightarrow \inf$).

Degeneration of the inclusion geometry affects cubic inclusions (e.g. rounding of edges) during the printing process. Therefore this geometric difference has also been simulated, by adding fillets to the inclusion edges with different radii.

Parameter optimization was performed to find the ideal values for the edge radius and contact stiffness. Simulations were performed with different edge radii (step size $10\,\upmu$m) and contact stiffness (step size $100\,\frac{\textrm{GN}}{\textrm{m}^3}$). The parameter set with the lowest accumulated deviation from the mean of the three measured sample sets was selected.

Inclusions with high volume fractions ($f_{inc}>85\%$), although not measured experimentally, were also simulated. Here the inclusions are interconnected and not separated within the matrix. The required volume fraction to connect the inclusions depends on their edge radius. Simulation steps beyond this value were chosen with a step width of $2.5\%$ of the inclusion volume.

#### Multimaterial homogenization

Multimaterial homogenization was performed to evaluate whether a model of ideal inclusion geometries (cubes and spheres), which does not consider inclusion-matrix interface properties, is still able to reproduce experimental findings. To parameterize this model the elastic compliance $\textbf{C}$, evaluated from the measurement values, was represented as a second-order tensor in Kelvin-Mandel notation. Second-order tensor representation was used for the Matlab implementation. The simulation was performed according to the Mori-Tanaka scheme, where inclusions are assumed to be in a homogeneous matrix material [38, 41, 42]. The volume increment of the simulation was set at $1\%$. The simulation models mixing scheme was adapted from Fritsch and Hellmich [8] and the Eshelby tensors from Yozo Mikata [43, 44]. For more details on multimaterial homogenization and the corresponding Hill- and Eshelby tensors in the implemented form, please refer to Appendix A.

## Results

**Fig. 2:**
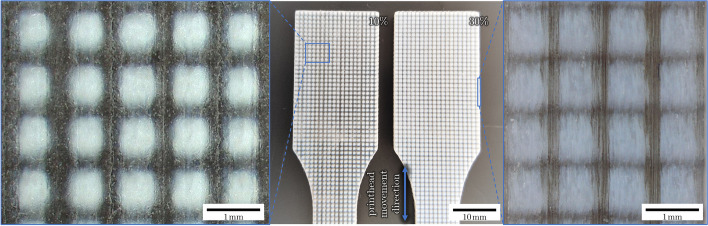
Left: Microscope image of a sample with a transparent *VeroClear* matrix and $f_{inc}=10\%$ cubical, grayish *RGD8530-DM* inclusions. On the surface are traces from the printer visible, small lines and droplet-circles, aligned to the moving direction of the print head (bottom to top). Center: Shoulder parts of two tensile testing specimen, type A according to EN ISO 3167. The left specimen has 10% opaque infill in a transparent matrix, while the right one has 30% infill. Right: Microscope image of a side view of a sample with an inclusion fraction of $f_{inc}=30\%$. The printing layers are visible (vertical lines)

No misprints occurred during production. In the transparent matrix, opaque inclusions are visible with the bare eye, as shown in Fig. 2, and the different inclusion volume fractions can be distinguished. In the direction, normal to the 3D printing plane, the transparent matrix allows to look through the material and gives a good view on the inclusions, however, traces from the printing are visible under the microscope. The side surfaces of the samples are much rougher than the top and bottom surfaces, and although inclusions are still visible, the otherwise transparent materials appear almost opaque.

### Tensile tests

The measured uniaxial Young’s modulus for the samples of the pure primary material *RGD8530-DM* was $1519.1\pm 39.9\,$MPa, and for *RGD8430-DM* it was $1773.8\pm 29.9\,$MPa. The two sample sets of pure *VeroClear*, originating from the two print jobs with different mixmaterials, yielded Young’s moduli of $2431.0\pm 29.6\,$MPa (printed together with *RGD8430-DM* samples) and $2444.6\pm 36.1\,$MPa (printed together with *RGD8530-DM* samples), with an insignificant deviation (two-sample t-test *p*-value for non-equal means $p = 0.5328$).

**Fig. 3:**
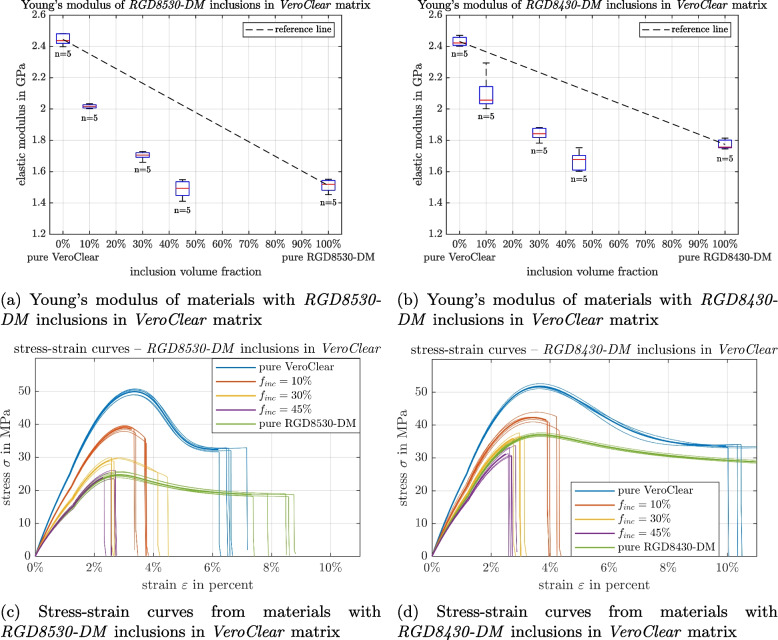
**a**, **b** Young’s moduli evaluated by tensile tests for samples made from pure materials and mixed materials, where cubical particles have been included to a *VeroClear* matrix. All matrix-inclusion composites have a lower Young’s modulus than the volume-averaged mean of the primary materials. A linear interpolation between the pure materials was added as a visual aid. **c**, **d** Stress-strain curves from tensile tests. Thin lines represent the individual measurements. Thick lines show the average value of the measurements. Vertical lines at the highest elongation of each curve mark the rupture of the samples

All tested multimaterial samples have a Young’s modulus below the volume-averaged mean of the primary materials. The multimaterials show a lower Young’s modulus than pure *VeroClear* samples, which further decreases with a higher content of the softer inclusion material. The results with *RGD8530-DM* and *RGD8430-DM* inclusions are plotted in Fig. 3. The detailed list of measured values is given in Appendix B.

Representative curves showing the technical stress over strain in samples from the print with *RGD8530-DM* are shown in Fig. 3c. The multimaterials rupture at earlier elongations than the samples made from pure materials. While the multimaterials ruptured at strains ranging from $2.3\%$ to $4.5\%$, the pure materials ruptured at strains ranging from $6.2\%$ to $22.7\%$, with the highest strains occurring in the pure *RGD8430-DM* samples.

**Fig. 4:**
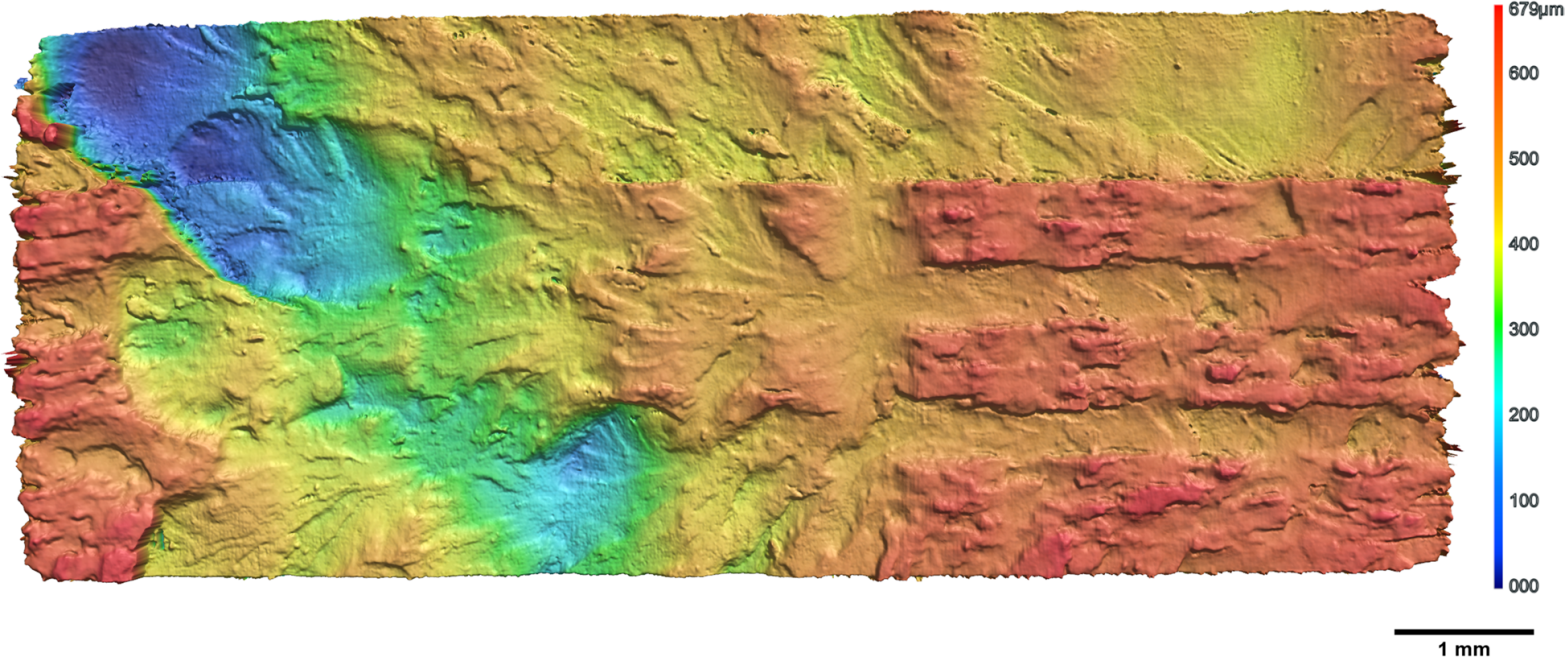
Profilometer scan of typical cross section from a ruptured sample with *VeroClear* matrix and $f_{inc}=30\%$ cubic *RGD8430-DM* inclusion materials. The color indicates the profiles height. On the right side the positions of the cubic inclusions are visible as elevations, indicating that the rupture propagates best at the inclusion-matrix interface

The samples with inclusions tore at the interface between inclusion and matrix material, indicating this cross section as the weakest part of the multimaterial. The height profile of a typical rupture area is shown in Fig. 4.

### OCT images

Since the used materials have a similar radiodensity, only little contrast can be achieved by computer tomography, but the inclusions with different colors are distinguishable by OCT imaging. For further image processing, planes of the materials containing inclusions have been investigated, and the selected region of interest (ROI) is the first layer of inclusions. Using OCT measurements with pure materials, a refractive index of $n=1.55$ for *VeroClear* and $n=1.5175$ for *RGD8530-DM* was found.

**Fig. 5:**
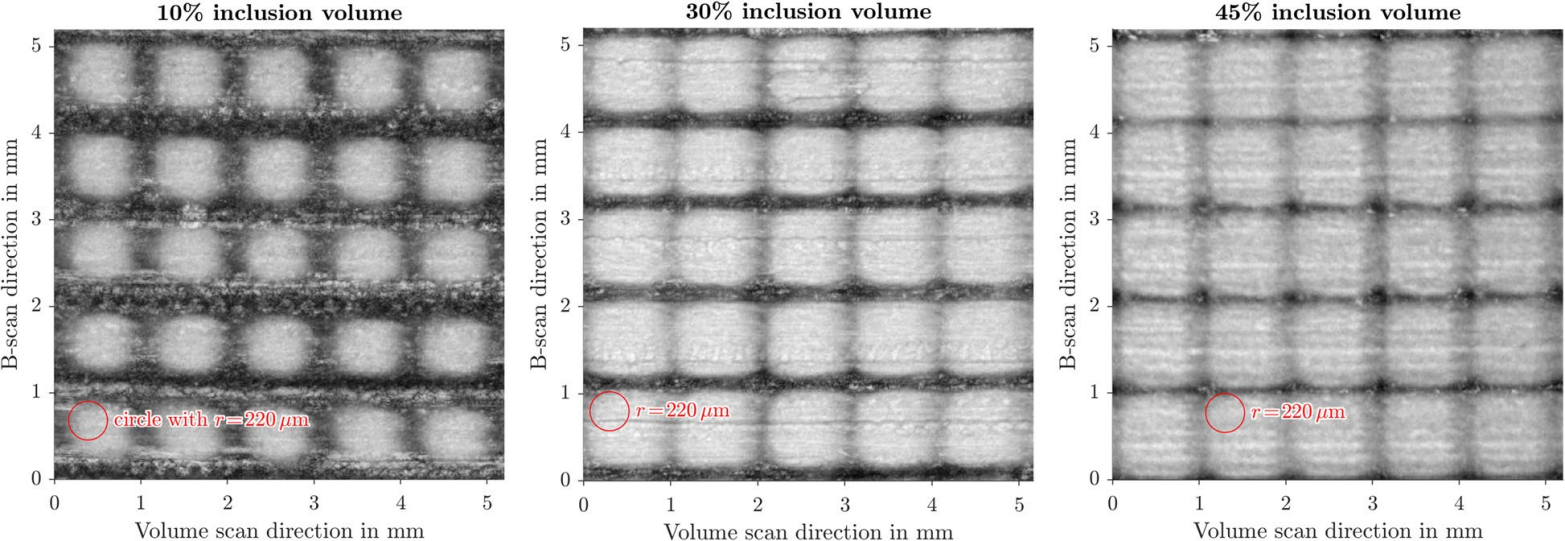
OCT-volume scan – Average intensity projection of the first inclusion layer of the multimaterial sample. These sample have volume fractions of $f_{inc}=10\%, 30\%, 45\%$ inclusion material (*RGD8530-DM*) shown in light gray, within a dark (*VeroClear*) matrix. For reference, a $r\!=\!220\,\upmu$m circle has been added, which corresponds to the inclusion edge radii used for the FEM simulation. The horizontal stripes are from the printing process, since the volume scan direction is the same direction as the print head moves

Shown in Fig. 5 is a projection of the volume scan, where the cubical inclusions can be seen in the matrix material. However, these cubes do not have a perfectly rectangular projection, but the rounded edges can be seen. Rounded edges with radii up to $r\!=\!400\,\upmu$m were found, while most edges have a radius from $r=200$ to $250\,\upmu$m.

From the image data, the planes of inclusions have been stacked to a single image and have been segmented to distinguish the inclusions and matrix more easily. The designed inclusion edge length for $1\!\times \!1\!\times \!1\,$mm unit cells with a volume fraction of $f_{inc}=30\%$ is $s_{inc}=669\,\upmu$m. Measurements showed that the cubes were smaller transversal to the printing direction, up to $\Delta l_p\!=\!-15\,\upmu$m. But the inclusions were larger, in the printing direction, up to $\Delta l_t\!=\!60\,\upmu$m. For comparison, the material jetting 3D printer has a resolution of $600\,$dpi, meaning that each voxel has a side length of $42.3\,\upmu$m.

**Fig. 6:**
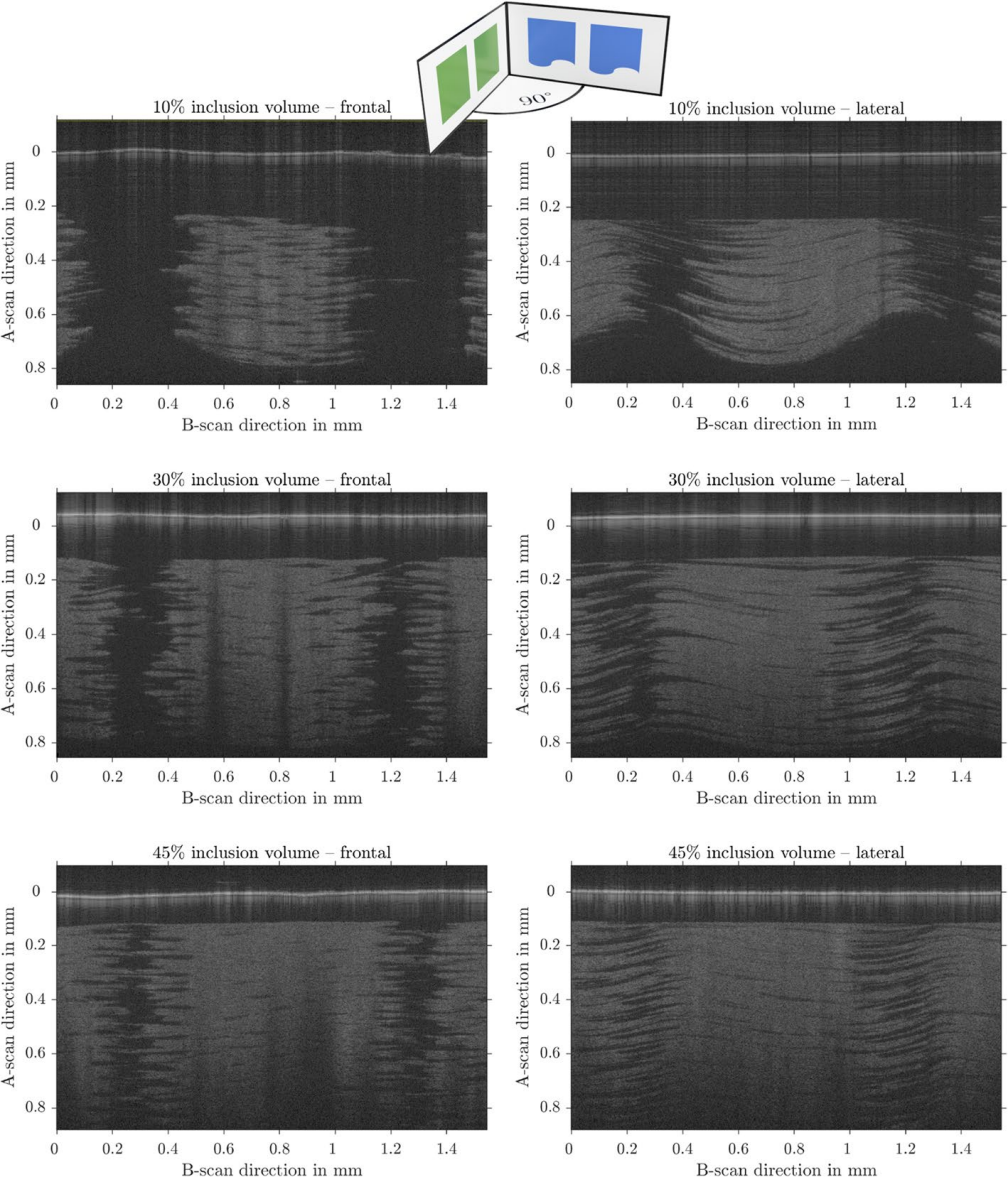
Micro OCT B-scans. The left views are cuts through the sample, perpendicular to the direction of printhead movement. The right panels show the same inclusions, but the view is rotated by $90^\circ$. The light objects are the inclusions to be seen on the dark background of the matrix material. The horizontal white bars on the top of the images are the surfaces of the samples. As shown on the right side, the inclusions are wavy in the direction of the print head movement. The first layers of the print are shown at the top of the image. Starting with the flat matrix layer, the waviness increases as the multimaterial layer is increased. All images show *RGD8530-DM* inclusions in *VeroClear* matrix. The volume fractions are $f_{inc} = 10\%$, $f_{inc} = 30\%$ and $f_{inc} = 45\%$ from top to bottom. The depth scale has been corrected from $1.484\,$mm optical distance to the geometric distance shown here

In Fig. 6 the inclusions are shown and the individual layers can be distinguished. It is important to notice that OCT images do not represent precise geometric distances, but instead optical distances that depend on the refractive indices of the materials and their transitions. However, these scans convey the information that between the layers there is a sharp transition between inclusions and matrix, but within the layers, the materials are more fringed, which can lead to different interface properties.

Also shown in Fig. 6 are the same inclusion, but with an orthogonal profile. A geometric deformation is visible, with a wavy surface on each cube. The waviness increases as more layers are formed during the printer’s operation. Not visible in this image is that the waviness of the layers decreases as the inclusions stop. After a few layers consisting purely of matrix material, the surface is planar again and the next inclusions start from flat again.

### Simulations

**Fig. 7:**
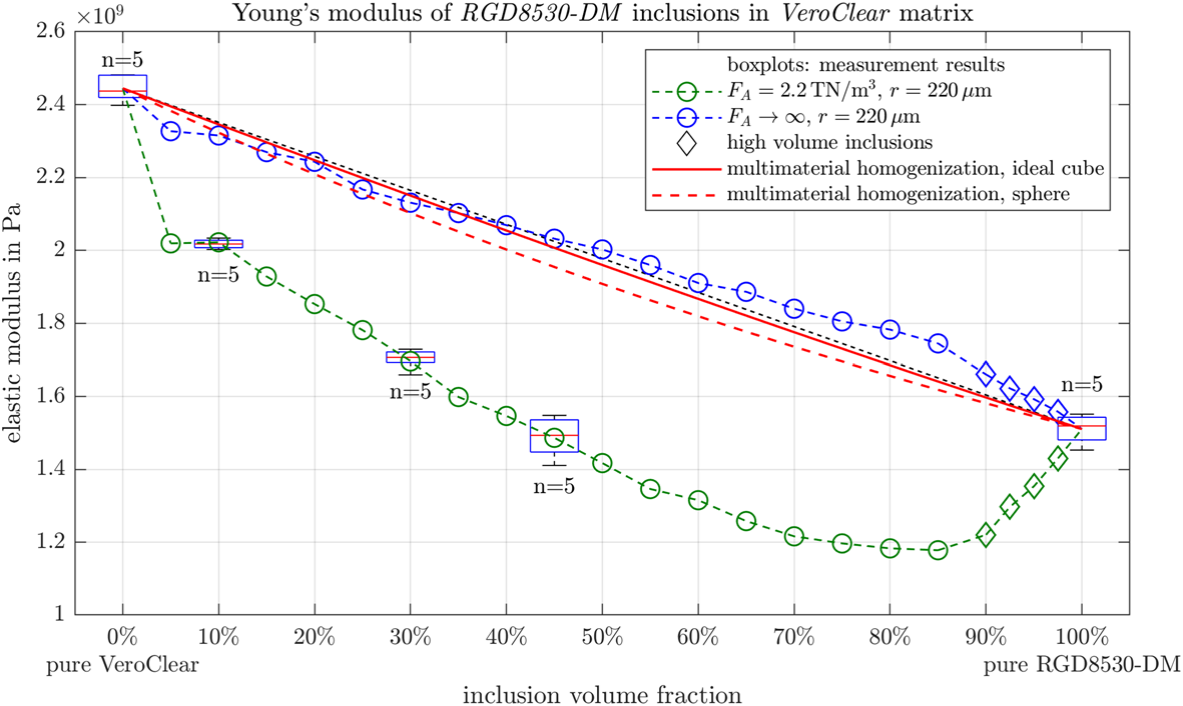
Young’s moduli of *RGD8530-DM* and *VeroClear* multimaterials, measurements are shown as boxplots. The FEM simulations for different mixture ratios are indicated. The contact stiffness between the inclusion and the matrix ($F_A$) is assumed to be $2.2\frac{\textrm{TN}}{\textrm{m}^3}$, and the edge radius $r=220\,\upmu$m. The second FEM simulation assumed ideally bonded contacts between inclusion and matrix. The red lines indicate the results from the multimaterial homogenization method. The reference line connects the mean values of the primary materials, showing a volume averaged mean value

Figure 7 shows the fit of the simulation of the matrix-inclusion composite to the measurement results. For *RGD8530-DM* inclusions in a *VeroClear* matrix, simulation results obtained using a contact stiffness of $2.2\frac{\textrm{TN}}{\textrm{m}^3}$ and a radius of $r=220\,\upmu$m that matched the OCT images, are within the interquartile range of the measurements. Accordingly, the inclusions will be in contact with each other at a volume fraction of $f_{inc,th}=88.96\%$ for $r=220\,\upmu$m. FEM simulations with ideal matrix-inclusion bonds are also shown and closely match the results from the multimaterial homogenization for perfectly cubic and spherical inclusions. The expected Young’s moduli from multimaterial homogenization are lower, but close, to the volume averaged mean of the pure materials (black dashed reference line), while the FEM simulation with ideally bonded contacts gives both slightly lower and higher values to the volume averaged mean.

**Fig. 8:**
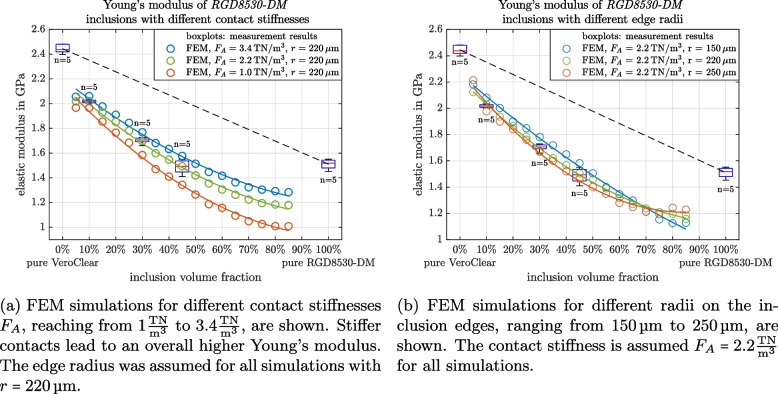
Effect on the Young’s moduli of *RGD8530-DM* and *VeroClear* multimaterials when the contact stiffness $F_A$ and edge radius *r* parameters are varied

Stiffer contact conditions between inclusion and matrix almost always mean that the entire material is also stiffer, with a translation of the overall stiffness at each volume inclusion ratio as shown in Fig. 8a. Also, a slight change in the inclination of the slope is observable. However, a change in the edge radii of the inclusions means that the behavior of the material mixtures differs with the curvature of the stiffness vs. inclusion fraction changing with changing radii Fig. 8b.

For multimaterials with *RGD8430-DM* inclusions, the same procedure was performed as well. According to the parameter optimization study, simulations also fit with an edge radius $r=220\,\upmu$m, however, a higher contact stiffness of $4.8\frac{\textrm{TN}}{\textrm{m}^3}$ was necessary to ensure a good fit.

### Poisson’s ratio

The measurement of Poisson’s ratio using the video extensometer provided measurements with high variability with standard deviations up to 0.08 (valid values for Poisson’s ratio $0\le \nu \le 0.5$). Therefore the median values of the pure materials were used as an input for the model. The values can be found in Appendix B Table 1.

**Fig. 9:**
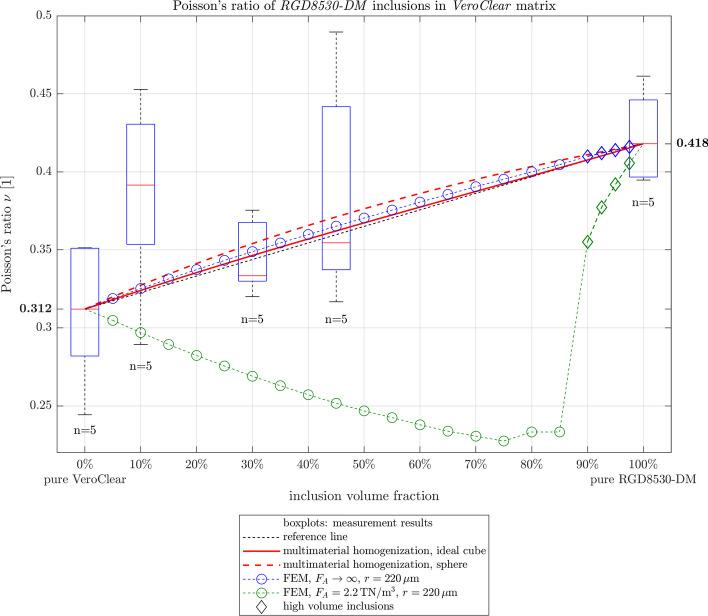
Poisson’s ratio from multimaterial homogenization and FEM simulations with ideally bonded and spring-loaded interface conditions. The Poisson’s ratio used for the cubic *RGD8530-DM* inclusions is $\nu _{RGD8530} = 0.418$, while the Poissons ratio for *VeroClear* matrix is $\nu _{VC} = 0.312$, as measured in the pure materials

The effects of different Poisson’s ratios were investigated by simulation. The results from the multimaterial homogenization show an arced behavior from one of the assumed Poisson’s ratios, known from this measurement or literature, for *VeroClear* to the Poisson’s ratio of *RGD8430-DM* $\nu _{RGD8430}=0.415$ and *RGD8530-DM* $\nu _{RGD8530}=0.418$. Depicted in Fig. 9 are the results for multimaterial homogenization with cubical inclusions in full lines and with spherical inclusions in dashed lines.

The Poisson’s ratios for the simulated multimaterials with ideally bonded interfaces and rounded cubical inclusions are in between the values of simulations with perfectly cubic or spherical inclusions. FEM simulations with either perfect cubic or spherical inclusions gave nearly the same results as the associated multimaterial homogenization (differences of the Poisson’s ratio $\ll 0.01$). Contrary to this, the simulations with spring-loaded interfaces show a decreasing Poisson’s ratio for higher inclusion fractions, away from the Poisson’s ratio of the inclusion material.

## Discussion

Results have shown that the material jetted multimaterials have Young’s moduli lower than the weighted average of the primary materials, in some cases even lower than that of the softer primary material. The relatively small standard deviations speak for the reliability of the measurements. Simulation results showed that well-tested methods to predict the properties of multimaterials, like multimaterial homogenization, cannot be applied easily, since the process of 3D printing introduces novel effects that influence mechanical material behavior.

While the results of the multimaterial homogenization agree with the FEM simulation with ideally bonded interfaces, a closer look at the samples (Fig. 2) reveals a zone where matrix and inclusion material is mixed. This mixture between the two material phases is most likely the cause of the difference between the two methods, and most likely the cause of the discrepancies between measurements and simulations under the assumption of ideal interface contacts. The fringed character of the interfaces can indeed be observed in the OCT images and confirms previous observations [33, 45]. The interface within a layer appears blurry, most likely because the jetted material droplets have a short time to blend before they are cured by UV light. This is also assumed the reason, why the cubes have radii on the edges, larger than the printer resolution.

Simulations with a finite contact stiffness, representing a spring-loaded interface, yield results with lower Young’s moduli. These simulations match better the measurement results, and choosing the right contact stiffness allows the prediction of the multimaterials properties. Especially when also the inclusion deformation (rounded edges) has been taken into account, simulations with a good fit to the measurement results could be established. The simulations show that the inclusion interface is essential to improve multimaterial predictions, and what the effect of varying inclusion geometries can be.

Although the simulation with a specific contact stiffness yields good results for Young’s modulus, it shows an unexpected behavior for the Poisson’s ratio, since this value is decreasing for higher inclusion contents, instead of rising to the Poisson’s ratio of the inclusion primary material. This is most likely due to the implementation of normal stiffness in the simulation software, as pulling on a spring, which represents the interfaces, is separating the surfaces of the inclusion and matrix. This causes a displacement that is not accounted for in the width change of the simulated geometry and therefore distorts the Poisson’s ratio. Possibly this model can be improved by implementing different stiffnesses, depending on the applied load and direction. For example finite stiffness values for tension, but infinity stiffness values ($F_a \rightarrow \inf$) for compression.

However, both the multimaterial homogenization, as well as FEM simulation with ideally bonded interfaces yield Poisson’s ratios whose trends coincide with the measured values. But changing the Poisson’s ratio in the simulation has only a minor impact on the Young’s modulus, which is in most applications the more important property to consider.

Since the literature provides many different values for the Poisson’s ratio of the primary material *VeroClear* [7, 46, 47], additional simulations, not shown in this article, were performed, where the Poisson’s ratio assumed for *VeroClear* was varied between the following values $\nu =[0.33;\, 0.35;\, 0.38;\, 0.41]$ in addition to the measured values of the pure materials. The Poisson’s ratio of *VeroClear* has a different impact on the simulation with ideally bonded interfaces (for rounded cubical *RGD8530-DM* inclusions: difference between $\nu _{VC}=0.33$ and $\nu _{VC}=0.41$: $13.9\,$MPa at $f_{inc}=10\%$; $13.0\,$MPa at $f_{inc}=50\%$; $2.17\,$MPa at $f_{inc}=85\%$), than on the simulation with a surface stiffness of $F_A = 2.2\,\frac{\textrm{TN}}{\textrm{m}^3}$ (difference between $\nu _{VC}=0.312$ and $\nu _{VC}=0.41$: $4.34\,$MPa at $f_{inc}=10\%$; $18.9\,$MPa at $f_{inc}=50\%$; $21.2\,$MPa at $f_{inc}=85\%$).

While the difference of the multimaterials Young’s moduli, depending on the matrix materials Poisson’s ratio, decreases at higher volume fractions for simulations with ideally bonded contacts, the simulations with spring-loaded interface conditions show the exact opposite behavior. However, for materials in the range of about $1\,$GPa, the change of a few MPa is insignificant.

Knowing the properties of one measured multimaterial, with a specific volume fraction of the inclusions, is sufficient to interpolate the contact conditions for a relatively simple model as outlined in this study, and therefore similar multimaterials with differing volume fractions can be predicted. However, we are still lacking a material model to predict mixed materials just from the properties of the primary materials, since we do not know how the interface conditions will turn out for new materials.

This can be seen particularly well when comparing the multimaterials with *RGD8530-DM* and *RGD8430-DM* inclusions. Here, the inclusion geometry depends mainly on the material jetting process, so the geometry, especially the edge radius, is similar. However, these different materials bond differently to the matrix material, therefore the contact stiffness varies.

Still, there are many further parameters that can be changed in FEM simulations, for instance, the tangential stiffness of the interface, since only variations in the normal stiffness were simulated in this study. Even more, when assuming an intermediate material between the matrix and the inclusion, there are suddenly many more parameters to be defined, like the position, geometry and contact conditions of the intermediate material, and of course the material properties itself. While it is easy to measure the properties of the primary materials, unfortunately, the contact stiffness or properties of an intermediate layer cannot be characterized with the same standardized tensile test, which in turn means, that these properties have to be estimated from indirect measurements. To emulate different interface properties, which are common to polyjetted materials, and especially pronounced with cubic inclusions, the boundary conditions for the contacts in layer (four lateral sides of the inclusions) can be assumed different to the conditions to the next layer (top and bottom sides).

In the field of multimaterial homogenization, there are also some approaches to model inclusions consisting of multiple layers [31, 32, 48, 49]. A possible improvement could be to represent the contact properties as an encapsulation of the inclusions.

Returning to the geometric deviations, OCT observed not only edge rounding but also non-flat, but wavy layers. Although it was beyond the scope of this study to account for this variation, it would be useful to investigate this effect in more detail. In particular, it should be determined whether the waviness of the layers in the printing direction occurs in all printing models, or whether it is only a peculiarity of this particular printing device. Depending on this, it may also be possible to determine under which conditions and geometries waviness occurs. If it turns out to be a common phenomenon, eventually caused by different shrinkage rates of the materials during photopolymerization [18], it will be necessary to investigate how such a geometry deviation can be predicted for multimaterials and considered in the material models.

Further observations from this and preliminary studies, which need further investigation, are the shelf time of the material cartridges. If the material even slightly changes its properties in the cartridge, although it is still usable for 3D printing, the resulting elasticity might change. This would have an impact on the repeatability and predictability of the material properties that have to be evaluated.

Also storing the materials after the print has not been investigated, as the samples were tested shortly after the production. However, it is quite conceivable that in applications where the elasticity of the material plays an important role, it should remain unchanged over a long period of time. Material aging, in particular the effect of composite materials and their contact points, still needs to be investigated.

It is reasonable to assume that this simulation concept applies to all polymers produced through material jetting. Since there are various highly elastic, rubber-like printing materials available, this expands the range of printable multimaterials further. Naturally, the parameters for contact stiffness would differ for other primary materials. Additionally, this FEM approach should work for other composites, regardless of how the individual components are arranged within the volume or whether more than two individual materials were used, since the geometry does not change the concept of non-ideal contact stiffness. A different arrangement of material phases will alter the overall behavior of the 3D-printed component. Especially concerning anisotropy, the freedom to choose inclusion geometries provides ample room for material design. This, in turn, could be highly valuable for creating as realistic as possible models for educational purposes in the medical field or for surgical planning.

An alternative arrangement of inclusions in a matrix-inclusion composite could also offer advantages in terms of tensile strength. This is because the experiments have shown that the interfaces between inclusions and the matrix are the weakest points of the material. By placing the inclusions in a way that eliminates the presence of a fracture plane, it becomes possible to achieve different fracture behaviors.

## Conclusion

In this study, material jetted matrix-inclusion composites, where the individual phases can be distinguished with the bare eye, were investigated for their mechanical and geometrical properties. This is the first time that this type of material jetted compound has been studied in detail, and conclusions drawn from this study can be applied to other geometries. Comprehensive material tests and OCT examinations were able to reveal some peculiarities of the polyjet printer, including deviations from the planned design. It has been observed that multimaterials depend not only on the starting materials, but also very much on the geometries and contact properties between the individual phases. This work confirms the findings that transition layers form between material phases, which have also been reported in several other articles. However, this is the first time that the effects of these deviations on material properties have been studied and incorporated into a simulation model.

For this kind of multimaterial, the Young’s modulus is not trivial to predict. Tuning the interface conditions between inclusion and matrix in the FEM simulation allows to estimate the properties of the resulting additively manufactured material. But these FEM simulations have a great variety of parameters to set, which already require knowledge of the multimaterials behavior. Since, to our best knowledge, no precise models exist, to predict the interface properties of 3D printed multimaterials, a minimum of empiric knowledge still is required as input for this model to achieve the desired material properties.

## Data Availability

Data will be made available on request.

## Appendix A Multimaterial homogenization

In Eq. 1 is shown how the estimated compliance $\textbf{C}^{est}$ of the multimaterial can be calculated from the compliance of the matrix material $\textbf{C}_0$. The individual material phases *r*, *s*, have their respective volume fractions $f_{r,s}$, compliances $\textbf{C}_{r,s}$ and Hill tensors $\textbf{P}_{0}^{r,s}$, where the geometrical information is inherited from the respective Eshelby tensor $\textbf{S}_{0}^{r,s}$. The unit tensor is denoted by $\mathbb {I}$.

1 $$\begin{aligned} \textbf{C}^{est} = \left( \sum _{r} f_r \textbf{C}_{r} \cdot [\mathbb {I}+\underbrace{\textbf{S}_0^{r} \cdot \textbf{C}_0^{-1}}_{\textbf{P}_0^{r}} \cdot (\textbf{C}_r-\textbf{C}_0)]^{-1}\right) \cdot \left( \sum _{s} f_s [\mathbb {I}+\underbrace{\textbf{S}_0^{s} \cdot \textbf{C}_0^{-1}}_{\textbf{P}_0^{s}} \cdot (\textbf{C}_s-\textbf{C}_0)]^{-1}\right) ^{-1} \end{aligned}$$

If there is only one inclusion phase to consider, Eq. 1, can be simplified to Eq. 2 for the inclusion phase *inc* and the matrix phase 0. Mind, that the Hill tensor $\textbf{P}$ considers the geometry of the inclusion, but the mechanical properties of the matrix material.

2 $$\begin{aligned} \textbf{C}^{est}{} & {} = \left( (1 - f_{inc}) \textbf{C}_{0} + f_{inc} \textbf{C}_{inc} \cdot [\mathbb {I}+{\textbf{P}_0} \cdot (\textbf{C}_{inc}-\textbf{C}_0)]^{-1}\right) \\{} & {} \quad \cdot \left( (1- f_{inc}) \mathbb {I} + f_{inc} [\mathbb {I}+{\textbf{P}_0} \cdot (\textbf{C}_{inc}-\textbf{C}_0)]^{-1}\right) ^{-1}\nonumber \end{aligned}$$

### Estimation of the mechanical Eshelby tensor

Although the numerical simulations used the second-order Eshelby tensor $\textbf{S}$ in Kelvin-Mandel notation, the mathematical derivation can be performed with the fourth-order Eshelby tensor $\mathbb {S}_{MnAb}$ representation, as given in Eq. 3.

3 $$\begin{aligned} \mathbb {S}_{MnAb}{} & {} = \frac{1}{8 \pi } \mathbb {C}_{iJAb} (I_{inMJ}+ I_{iMnJ}), \quad \text {with}\ M,n,A,b,i,J \in \{1,2,3\}\end{aligned}$$

4 $$\begin{aligned} \varvec{K}_{MJ}(\vec {x}){} & {} = \mathbb {C}_{pMJq} \cdot x_p \cdot x_q, \quad \text {with}\ M,J,p,q \in \{1,2,3\} \end{aligned}$$

5 $$\begin{aligned} I_{\textit{abcd}}{} & {} = \int _{|y|=1}y_\textit{a} y_\textit{b} \varvec{K}_{\textit{cd}}^{-1}(y_1, y_2, y_3) d\vec {y},\quad \text {with}\ a,b,c,d \in \{1,2,3\}\\ \text {with}\; \vec {y}{} & {} = y_i \; : \, i \in \{1,2,3\} \nonumber \end{aligned}$$

The second-order tensor $\varvec{K}_{MJ}$, dependent from the directions in the three dimensional space $\vec {x}$, from Eq. 4 can be stated as in Eq. 7, assuming a compliance tensor $\textbf{C}$ as in Eq. 6, where it is given in Kelvin-Mandel notation. Together with the inclusions geometry, parameterized with the three dimensional space coordinates $\vec {y}$, the surface integrals $I_{abcd}$ can be solved, which are required to estimate the Eshelby tensor $\mathbb {S}$.

6 $$\begin{aligned} \textbf{C} = \left( \begin{array}{cccccc} c_{11} &{} c_{12} &{} c_{13} &{} 0 &{} 0 &{} 0 \\ c_{21} &{} c_{22} &{} c_{23} &{} 0 &{} 0 &{} 0 \\ c_{31} &{} c_{32} &{} c_{33} &{} 0 &{} 0 &{} 0 \\ 0 &{} 0 &{} 0 &{} 2\, c_{44} &{} 0 &{} 0 \\ 0 &{} 0 &{} 0 &{} 0 &{} 2\, c_{55} &{} 0 \\ 0 &{} 0 &{} 0 &{} 0 &{} 0 &{} 2\, c_{66} \end{array}\right) \end{aligned}$$

7 $$\begin{aligned} \varvec{K}_{MJ}(\vec {x}) = \left( \begin{array}{ccc} c_{11}\,{x_{1}}^2+c_{66}\,{x_{2}}^2+c_{55}\,{x_{3}}^2 &{} x_{1}\,x_{2}\,\left( c_{12}+c_{66}\right) &{} x_{1}\,x_{3}\,\left( c_{13}+c_{55}\right) \\ x_{1}\,x_{2}\,\left( c_{21}+c_{66}\right) &{} c_{66}\,{x_{1}}^2+c_{22}\,{x_{2}}^2+c_{44}\,{x_{3}}^2 &{} x_{2}\,x_{3}\,\left( c_{23}+c_{44}\right) \\ x_{1}\,x_{3}\,\left( c_{31}+c_{55}\right) &{} x_{2}\,x_{3}\,\left( c_{32}+c_{44}\right) &{} c_{55}\,{x_{1}}^2+c_{44}\,{x_{2}}^2+c_{33}\,{x_{3}}^2 \end{array}\right) \end{aligned}$$

The corresponding second-order Hill tensor $\textbf{P}$ can be calculated from the second-order Eshelby tensor in Kelvin-Mandel notation $\textbf{S}$ and the material properties $\textbf{C}$, according to Eq. 8.

8 $$\begin{aligned} \textbf{P} = \textbf{S} \cdot \textbf{C}^{-1} \end{aligned}$$

## Appendix B Sample materials

All used materials are commercially available, made for the use in the *Connex3 Objet500* polyjet printer and products of Stratasys Ltd. (Minnesota, USA).

- *VeroClear* is a transparent rigid material, also known as *RGD810*.
- *VeroPureWhite* is an opaque rigid material, also known as *RGD837*.
- *TangoPlus* is a transparent flexible material, also known as *FLX930*.
- *TangoBlackPlus* is a flexible, opaque black material, also known as *FLX980*.
- *RGD8530-DM* is a “digital material” mixed in th printer during fabrication from *VeroPureWhite* and *TangoBlackPlus*.
- *RGD8430-DM* is a “digital material” mixed in the printer during fabrication from *VeroPureWhite* and *TangoPlus*.

**Table 1:** Material properties obtained by measurements

Material	Young’s modulus mean value [MPa]	Young’s modulus median value [MPa]	Shapiro-Wilk significance^a^ $\varvec{p}_{\varvec{sw}}$ of the Young’s modulus	median Poisson’s ratio $\varvec{\nu }$
Vero Clear, printed together with RGD8430-DM samples	2431 ± 29.6	2422	0.492	0.318
Vero Clear, printed together with RGD8530-DM samples	2445 ± 36.1	2437	0.428	0.312
RGD8430-DM	1774 ± 29.9	1757	0.219	0.415
RGD8530-DM	1511 ± 39.9	1519	0.704	0.418
VeroClear matrix, 10% cubic RGD8430-DM inclusions	2098 ± 115	2056	0.099	0.337
VeroClear matrix, 30% cubic RGD8430-DM inclusions	1842 ± 39.9	1842	0.564	0.350
VeroClear matrix, 45% cubic RGD8430-DM inclusions	1666 ± 61.1	1677	0.568	0.389
VeroClear matrix, 10% cubic RGD8530-DM inclusions	2017 ± 12.5	2017	0.923	0.392
VeroClear matrix, 30% cubic RGD8530-DM inclusions	1703 ± 26.5	1706	0.309	0.333
VeroClear matrix, 45% cubic RGD8530-DM inclusions	1489 ± 55.6	1493	0.796	0.355

The Shapiro-Wilk test assumes the sample as normally distributed, if the desired statistical significance $\alpha \le p-$value
